# Preparation, Characterization, and Antibacterial Activity of Various Polymerylated Divalent Metal-Doped MF_2_O_4_ (M = Ni, Co, Zn) Ferrites

**DOI:** 10.3390/polym17091171

**Published:** 2025-04-25

**Authors:** Enas AlMatri, Nawal Madkhali, Sakina Mustafa, O. M. Lemine, Saja Algessair, Alia Mustafa, Rizwan Ali, Kheireddine El-Boubbou

**Affiliations:** 1 Department of Chemistry, College of Science, University of Bahrain, Bahrain 32038, Bahrain; enas.almatrii@gmail.com; 2 Department of Physics, College of Science, Imam Mohammad Ibn Saud Islamic University (IMISU), Riyadh 11623, Saudi Arabia; namadkhali@imamu.edu.sa (N.M.); mamamin@imamu.edu.sa (O.M.L.); saja.algessair@gmail.com (S.A.); 3 Department of Biology, College of Science, University of Bahrain, Bahrain 32038, Bahrain; smustafa@uob.edu.bh; 4 Department of Physics, College of Science, University of Bahrain, Bahrain 32038, Bahrain; amkhan@uob.edu.bh; 5 Medical Research Core Facility and Platforms (MRCFP), King Abdullah International Medical Research Center (KAIMRC), King Saud bin Abdulaziz University for Health Sciences (KSAU-HS), King Abdulaziz Medical City (KAMC), National Guard Health Affairs (NGHA), Riyadh 11481, Saudi Arabia; aliri@kaimrc.edu.sa

**Keywords:** iron oxide nanoparticles, magnetite, doped iron oxides, doped ferrites, anti-bacterial, inhibition, *E. coli*, gram-negative bacteria

## Abstract

The continuous discovery of novel effective antibacterial agents using nano-based materials is of high significance. In this study, we utilized Polymerylated divalent-metal-doped ferrite nanoparticles (PMFe_2_O_4_ NPs) and studied their antibacterial inhibition effects. Different panels of PVP- and PEG-coated metal-doped MFe_2_O_4_ (M ≅ Co, Ni, and Zn) were prepared via the *Ko-precipitation Hydrolytic Basic* (KHB) methodology and thoroughly analyzed using TEM, XRD, FTIR, and VSM. The as-synthesized doped ferrites displayed stable quasi-spherical particles (7–15 nm in size), well-ordered crystalline cubic spinel phases, and high-saturation magnetizations reaching up to 68 emu/g. The antibacterial efficacy of the doped ferrites was then assessed against a Gram-negative *E. coli* bacterial strain. The results demonstrated that both metal doping and polymer functionalization influence the antimicrobial efficacies and performance of the ferrite NPs. The presence of the PVP polymer along with the divalent metal ions, particularly Co and Ni, resulted in the highest antibacterial inhibition and effective inactivation of the bacterial cells. The antibacterial performance was as follows: PVP-CoFe_2_O_4_ > PVP-NiFe_2_O_4_ > PVP-ZnFe_2_O_4_. Lastly, cell viability assays conducted on human breast fibroblast (HBF) cells confirmed the good safety profiles of the doped ferrites. These interesting results demonstrate the distinctive inhibitory features of the biocompatible metal-doped ferrites in enhancing bacterial killing and highlights their promising potential as effective antimicrobial agents, with possible applications in areas such as water disinfection, biomedical devices, and antimicrobial coatings.

## 1. Introduction

Pathogenic infectious diseases pose a significant challenge to global public health and have long been leading contributors to worldwide mortality [1]. Despite recent advancements in medicine, the development of various antibiotic classes, improved hygiene, and prevention strategies, there has been a concerning renaissance of new pathogenic diseases that remain difficult to treat [2]. Microbes and bacteria, with their capacity to constantly evolve, have developed resistance to many currently prescribed antibiotics. One famous example is *Escherichia coli* (*E. coli*)—one of the most concerning drug-resistant bacterial strains—which has led to a high death percentage, particularly in low-income countries [3]. It has been estimated that by 2050, 10 million deaths worldwide will be attributed to resistant pathogens, with a USD 100 trillion loss in GDP if direct actions are not taken [4,5]. The escalating prevalence of antimicrobial resistance has triggered a global healthcare alarm, highlighting the urgent need for the development of novel and cost-effective antimicrobic strategies. Thus, nanomaterials exhibiting promising antimicrobial properties have garnered increasing attention [6,7].

Magnetic iron oxide nanoparticles (MNPs) have been recently explored for various environmental applications, including water treatment [8], microbial removal [9], sensing and detection of pathogenic bacteria [10], as well as antibacterial inhibition [11,12,13]. Among these, magnetite (Fe_3_O_4_) NPs have been especially promising due to their low toxicity, unique and tunable magnetic properties, and easy surface functionalization [14,15]. Nevertheless, the respective biological activity of Fe_3_O_4_ NPs is closely related to their particle size and surface coating. Polymers are, thus, required to modify such MNPs to play a better role in practical antibacterial applications. It is crucial to coat ferrite NPs with suitable polymers to enhance their biocompatibility and stability in biological media, while also minimizing aggregation and precipitation in solutions [16]. This improved stability and dispersibility is critical for antimicrobial applications, where the NPs should remain well-dispersed and stable in biological media. Among the numerous utilized polymers, polyvinylpyrrolidone (PVP) and polyethylene glycol (PEG) are well-known, non-ionic, water-soluble polymers that are frequently employed in biological and pharmaceutical applications as dispersing agents, binders, and stabilizers. Consequently, the synthesis of high-quality colloidal MNPs coated with these polymers and the evaluation of their specific antimicrobial efficacies are highly demanded.

On the other hand, doping MNPs with divalent metal atoms has been shown to enhance the antimicrobial activities, where doped ferrites (MFe_2_O_4_; where M = Cobalt (Co), Nickel (Ni), or Zinc (Zn) etc.) have been regarded as excellent materials in this regard [17,18,19,20]. Doping involves the controlled incorporation of foreign metal elements into specific sites of the crystal lattice, resulting in modifications to the material’s physicochemical, biological, and antimicrobial properties. Divalent-doped spinel ferrites can enhance the stability and reactivity of MNPs compared to their bare counterparts, making them more suitable for various bacterial applications [21]. The presence of divalent metals can affect the oxidation state of the NPs, potentially enabling improved redox reactions or electron transfer processes. For instance, very good antibacterial inhibition abilities of Ni-ferrites (Ni-Fe_2_O_4_) NPs were reported [22,23], where their ability to generate of reactive oxygen species (ROS) induce oxidative stress and damage in the bacterial cells, enhancing their antimicrobial efficacy [24]. On the other hand, Co-ferrite (CoFe_2_O_4_) NPs have gained prominence as effective antibacterial agents due to their exceptional physical features, including high coactivity, excellent chemical stability, large magneto-strictive coefficient, and moderate saturation magnetization [25,26]. Spinel Zn-ferrites (ZnFe_2_O_4_) were also applied successfully either alone [27] or mixed with Co or Ni in limelight to find their use as antibacterial applications [28]. Although the antibacterial mechanisms of NPs are poorly understood, the most accepted mechanisms include oxidative stress induction and metal ion release. The high surface areas and small sizes of ferrite NPs allow them to efficiently interact with the surface structures of bacteria, expediting their uptake by bacterial cells. Many previous studies have shown that NPs exhibit enhanced antibacterial activity by penetrating bacterial cells, disrupting ribosomal function to inhibit protein synthesis, or binding to bacterial DNA, which ultimately causes cellular damage and death [18,29].

Doped MNPs, particularly divalent-metal-doped ferrites, provide several unique advantages over both traditional antibiotics and other known antimicrobial nanomaterials, as follows: (a) Tunable activity: Doping allows precise control over surface charge density, band gap, and crystal structure, all of which directly influence ROS generation and antibacterial potency. This tunability is not possible with single-element NPs like Ag NPs or with conventional antibiotics; (b) Dual functionality: Unlike antibiotics, which rely on biochemical interactions with bacteria (which have developed increased resistance due to excessive use of these drugs) [3], doped ferrites exert both physical (membrane attachment and disruption) and chemical (oxidative stress via ROS) mechanisms. This makes them less prone to bacterial resistance [18]; (c) Magnetic responsiveness: Their magnetic nature allows external control—enabling easy magnetic separation—which is not feasible with Ag or Cu NPs; (d) Cost and stability: Iron oxide is both abundant and inexpensive, and doping with small amounts of metals adds functionality without dramatically increasing cost—unlike the noble metal NPs. Importantly, in contrast to traditional antibacterial agents that often exhibit high cytotoxicity to both bacterial and mammalian cells, doped ferrites offer a selective mechanism of action. Their antibacterial activity is primarily driven by membrane disruption and ROS generation, which effectively target bacterial structures without harming human mammalian cells [27]. Moreover, biocompatible coatings, such as PEG or PVP, further reduce nonspecific interactions with human cells while preserving antibacterial potency. This unique combination of efficacy and safety makes doped ferrites a promising alternative to normal cytotoxic agents like Ag NPs. Taken together, doped ferrite NPs offer a modular, multi-mechanism, and magnetically responsive platform for antibacterial applications, with reduced risk of toxicity and resistance—a profile unmatched by current conventional antimicrobial agents. Nonetheless, many ferrite NPs reported in the literature are synthesized without appropriate surface coatings, rendering them unsuitable for biomedical applications due to reduced biocompatibility, limited aqueous dispersibility, and increased susceptibility to oxidation. Moreover, most studies usually focus on only one doped ferrite formulation rather than comparing different formulations and their effects on antibacterial inhibition activities.

In this study, we synthesized a series of PVP- and PEG-coated metal-doped MFe_2_O_4_ ferrite NPs (where M ≅ Co, Ni, or Zn) using our *Ko-precipitation Hydrolytic Basic* (KHB) method and assessed their antibacterial efficacy. The physicochemical, morphological, compositional, and magnetic properties of the doped ferrites using various spectroscopic and electronic techniques were thoroughly analyzed. As proof of concept, the antibacterial activity of the different doped ferrites was evaluated against *E. coli* under different conditions, describing their minimum inhibitory concentrations (MIC) and bacterial growth patterns. To the best of our knowledge, this is the first comprehensive study examining the antibacterial inhibition properties of various PVP- and PEG-coated metal-doped ferrites, underscoring their promising potential as effective antimicrobial agents.

## 2. Experimental Section

### 2.1. Materials and Methods

All chemicals and solvents were obtained from commercial suppliers and used as supplied without further purification. Iron(III) chloride hexahydrate (FeCl_3_.6H_2_O), iron(II) sulfate heptahydrate (FeSO_4_.7H_2_O), Cobalt(II) chloride hexahydrate (CoCl_2_.6H_2_O), Nickel(II) chloride hexahydrate (NiCl_2_.6H_2_O), Zinc(II) chloride hexahydrate (ZnCl_2_.6H_2_O), polyethylene glycol (PEG Mw~4000), polyvinylpyrrolidone (PVP Mw ~ 40,000), and 28% ammonium hydroxide (NH_4_OH) were all purchased from Fisher Scientific (Waltham, MA, USA)and Sigma Aldrich (Saint Louis, MO, USA). All reactions were carried out under inert nitrogen atmosphere. For the biological bacterial assays, Nutrient broth, Luria Bertani (LB) broth, and NaCl were purchased from Merk and Sigma Aldrich. For the cytotoxicity assay, Dulbecco’s Phosphate-Buffered Saline (DPBS), Phosphate-Buffered Saline (PBS), Advanced Dulbecco’s Modified Eagle Medium (DMEM), Phenol-red free DMEM, and Fetal Bovine Serum (FBS) were all purchased from Invitrogen. Patient-derived primary human breast fibroblast (HBF) cells were isolated from the patient after appropriate consent and Institutional Review Board (IRB) approval. The cells were maintained in DMEM supplemented with 10% FBS, 50 units/mL penicillin, 50 µg/mL streptomycin (Gibco), and 2 mM L-glutamine (Gibco) at 37 °C in a humidified 5% CO_2_ atmosphere.

#### Characterization

X-ray diffraction (XRD) spectra were analyzed using Rigaku Uitima IV (Rigaku, Tokyo, Japan) equipped with a Cu-Kα radiation source (0.15418 nm) with an angle ranging from 20° to 80°, and the crystal structure parameters were obtained through Rietveld analysis. Transmission electron microscopy (TEM) images were collected on a JEOL-JEM 1400 operating at 120 kV using a Gatan camera with Digital Micrograph Imaging software (JEOL, Pleasanton, CA, USA). The samples were prepared by depositing 1 μL of the doped MFe_2_O_4_ NPs onto a 400-mesh Formvar/Carbon-supported copper grid. Fourier transform infrared (FTIR) spectra (400–4000 cm^−1^) were recorded as KBr pellet forms using Shimadzu IRAffinity-1. Magnetic characterizations were performed using a vibrating sample magnetometer (VSM) with 1.8 T magnets at an ambient temperature.

### 2.2. Preparation of Polymer-Coated MFe_2_O_4_ NPs

All MNPs were synthesized using our previously established KHB method [30,31,32]. FeCl_3_·6H_2_O (0.30 g) was dissolved in 10 mL of water containing PVP or PEG (0.2 g), and the mixture was stirred for a few minutes at 80 °C under nitrogen conditions. A solution of Fe^2+^:M^2+^ (0.1 g:0.1 g) in water was then injected into the above solution. Ammonium hydroxide (28% NH_4_OH, ~3 mL) was gradually introduced, causing the solution to turn a black-brick color, indicating the formation of doped MNPs. Stirring continued for an additional 3 hrs. The NP suspensions were then purified by centrifugation, followed by several washes with isopropanol, ethanol, and water. Finally, the obtained MNPs were re-dispersed in water to obtain stable aqueous dispersions of polymer-coated doped MFe_2_O_4_ NPs. For the preparation of Fe_3_O_4_ NPs, the same procedure was followed using 0.2 g of Fe^2+^.

### 2.3. Antibacterial Inhibition Experiments

The test organism, Gram-negative *Escherichia coli* (*E. coli*), was grown separately in 50 mL sterilized Luria Bertani (LB) broth medium and kept in a shaker incubator at 37 °C for 18 h (overnight incubation). On the subsequent day, the *E. coli* cultures were transferred at the rate of 1% in 50 mL LB broth kept in conical flasks. Various MFe_2_O_4_ NPs (25 mg, final concentrations 0.5 mg/mL) were placed into each flask, leaving one as a control to track the normal growth of the bacterial cells without NPs. The flasks were shaken at 180 rpm at 37 °C in a shaker incubator. Optical density measurements from each flask were taken every hour to record the growth of the microbes with a spectrophotometer set at 600 nm. The spectrophotometer was blanked with aqueous NP solutions without bacterial cells. The growth rate of microbial cells interacting with the NPs was determined from a plot of the log of the optical density vs. time, as reported earlier [33]. To determine the minimum inhibitory concentration (MIC) of the MFe_2_O_4_ NPs, the broth microdilution method was used. Briefly, bacterial suspensions were prepared in 0.85% saline in a concentration of around 10^8^ cells/mL, as adjusted to 0.5 McFarland standards. The MFe_2_O_4_ NPs were diluted 1:1 fold through 96-well microplates, making dilutions from 1000 to 15.6 µg/mL. After the addition of the bacterial suspensions to the wells, the microplates were incubated at 37 °C for 18–24 h. After incubation, the OD_600_ of the bacterial growth was measured with a microplate reader. The growth percentage of *E. coli* and the minimum concentration that completely inhibited bacterial growth was accordingly determined.

### 2.4. Cell Viability and Proliferation Assay

HBF cells were grown in advanced DMEM containing 10% fetal bovine serum, 1% L-glutamine, and 1% antibiotics (Pen/Strep). The cells were cultured in a 96-well plate overnight before treatment at a density of 10,000 cells per well. HBF cells were then exposed to undoped and doped PVP-coated MFe_2_O_4_ NPs (M = Fe, Zn, Ni, or Co) at various increasing concentrations ranging from 0 to 100 µg/mL to determine their cytotoxic effect. An MTT assay was used to determine cell viability after a 48 h incubation period. The MTT assay followed the standard protocol shown in our previous works, and the absorbance was read at 560 nm using Molecular Devices Spectra Max Plus 384 Spectrophotometer (Molecular Devices, San Jose, CA, USA). Background readings were collected at 620 nm. Absorbance readings were normalized and expressed as a relative percentage. The experiments were carried out in quadruplicates, analyzed using Microsoft excel, and data are presented as mean values ± standard deviations (SD).

## 3. Results and Discussion

### 3.1. Preparation and Characterization of Polymer-Coated Ferrites

**Figure 1:**
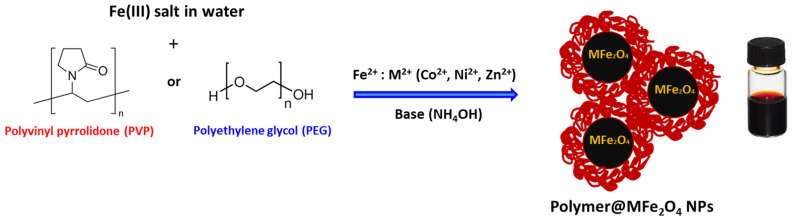
Schematic illustration for the preparation of polymer-coated MFe_2_O_4_ ((M = Co, Ni, Zn) NPs using the *K*o-precipitation *H*ydrolytic *B*asic (KHB) approach.

**Figure 2:**
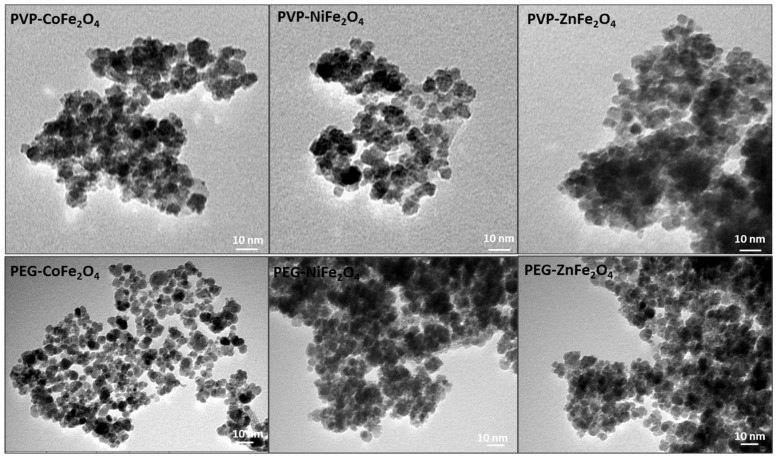
TEM images of the various PVP- and PEG-coated metal-doped MFe_2_O_4_ nanoferrites.

**Figure 3:**
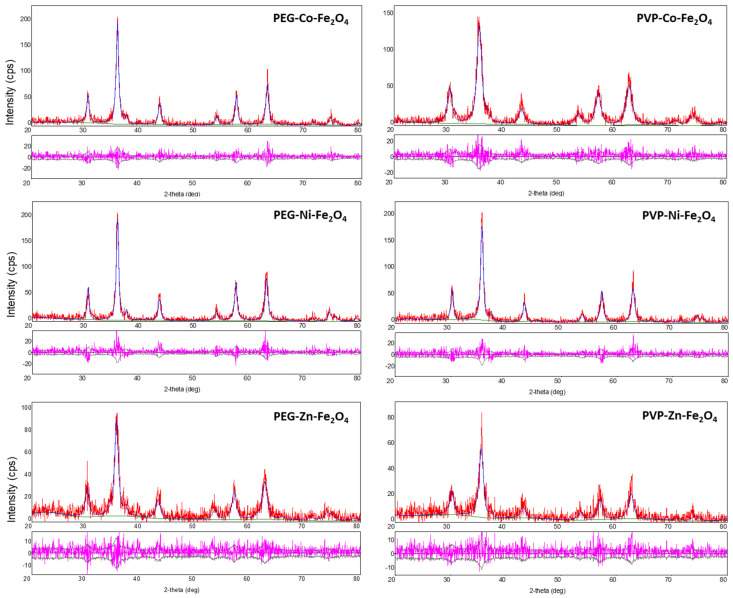
XRD patterns of the various PVP- and PEG-doped ferrites along with Rietveld refinement profiles. The red line represents the observed experimental data, the blue line corresponds to the Rietveld refinement fit, and the lower pink curve illustrates the difference between the observed and calculated data at each step.

PVP- and PEG-coated MFe_2_O_4_ ferrites were prepared following our recently developed *K*o-precipitation *H*ydrolytic *B*asic (KHB) methodology [30] (Figure 1). The approach relies on sequential basic hydrolytic *in situ precipitation* (*Ko-precipitation*) of inexpensive metal salts (Fe^3+^ and M^2+^ where M^2+^ = Fe^2+^, Co^2+^, Ni^2+^, or Zn^2+^) compartmentalized by stabilizing polymers (i.e., PEG or PVP) in the presence of a base (NH_4_OH). This allowed the formation of stabilized polymer-coated MFe_2_O_4_ ferrite NPs well-dispersed in aqueous solutions. TEM clearly indicated the ultrasmall sizes of all ferrite NPs, with average sizes of ~7–15 nm, as indicated in the images (shown for PVP-coated ferrites) (Figure 2). To elucidate the crystallite structure and identify the phase of the obtained ferrites, XRD was performed. The XRD patterns of PVP- and PEG-coated MFe_2_O_4_ samples are shown in Figure 3. The results showed pure crystalline single-phase inverse spinel structures with well-defined and sharp peaks at (220), (311), (400), (422), (511), (440), and (533). These patterns confirmed the formation of pure MFe_2_O_4_ cubic spinel phases with space group Fd3m for both PVP- and PEG-coated NiFe_2_O_4,_ CoFe_2_O_4_, and ZnFe_2_O_4_, which matches well with JCPDS #01-071-3850, #01-074-6402, and #01-071-5149, respectively. Both polymer-coated CoFe_2_O_4_ and NiFe_2_O_4_ exhibit inverse spinel structures, where Co^2+^ and Ni^2+^ ions preferentially occupy octahedral sites, while Fe³^+^ ions occupy tetrahedral ones. The lowest crystallinity was observed for polymer-coated ZnFe_2_O_4_, likely due to the larger ionic radius of Zn^2+^, leading to a less ordered structure [34]. Crystallite size, microstrain, and lattice parameters for all polymer-doped ferrite samples were determined through Rietveld refinement analyses, as detailed in Table 1. The data clearly indicate that doping with M^2+^ ions did not significantly alter the crystal structure or lattice parameters. This outcome was anticipated, as substituting Fe^2+^ ions with small amounts of M^2+^ ions (Fe^2+^:M^2+^; 1:1) is unlikely to induce geometric distortion in the unit cell, as has been previously observed by us and others [30,35].

**Figure 4:**
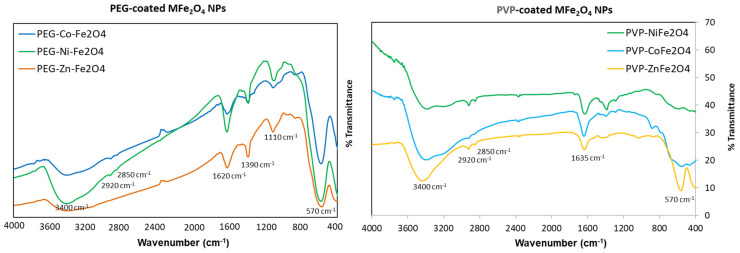
FTIR spectra for the different polymer-coated MFe_2_O_4_ NPs clearly showing the successful polymers coating the nanoferrites.

Next, FTIR analysis was conducted to confirm the successful formation of polymer coatings on MNPs, as depicted in Figure 4. The presence of iron oxide (Fe_3_O_4_) in the core was evident by the Fe−O stretching vibrations, which appeared as one broad peak of ~570 cm^−1^ for the MFe_2_O_4_ due to metal doping of the crystallites [30]. The distinct clear peaks observed at 2850 and 2920 cm^−1^ correspond to the symmetric and asymmetric C–H stretching vibrations of PVP and PEG coatings, respectively. The broad peak at ~3400 cm^−1^ is attributed to the O–H stretching vibration of hydroxyl groups (-OH) present on MNPs. The prominent peaks at 1635 cm^−1^ in the FTIR spectra of PVP-coated ferrite NPs corresponds to the stretching vibration of the C=O carbonyl group, which is slightly shifted from the typical 1660 cm^−1^ observed in pure PVP. This indicates functionalization of the MNPs with PVP through intermolecular hydrogen bonding between the C=O of PVP and the protonated hydroxyl groups on the MNP surfaces, confirming the interaction between PVP and metal oxide surfaces [36]. Moreover, PVP is known to adsorb onto ferrite nanocrystals through coordinative bonds between the pyrrolidone molecules and metal ions [37]. For PEG coating, on the other hand, the presence of strong peaks at ~1110 cm^−1^ can be associated to the distinctive C-O-C stretching vibrations within the PEG backbone. The absorption bands around 1620 cm^–1^ originated from stretching and deformation vibration hydroxyl groups connected to the surface of NPs. The -CH groups scissoring in-plane bending were also observed at 1390 cm^−1^ [38,39]. All of these findings collectively validate the successful functionalization of the ferrite NPs with the respective polymers.

### 3.2. Magnetic Properties

**Figure 5:**
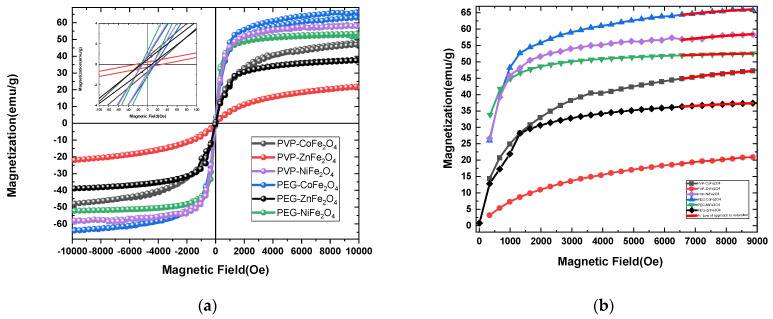
(**a**) Magnetization hysteresis curves for the different PVP- and PEG-coated MFe_2_O_4_ ferrites at room temperature (inset: low field −400–400 Oe); (**b**) Fitting of law of saturation approach.

To assess the magnetic properties of the polymer-coated doped ferrites, field-dependent magnetization measurements were performed. These measurements are essential for understanding the magnetic behavior of MNPs, particularly in biomedical applications where magnetic responsiveness is crucial. It is to be noted that the magnetic properties of polymer-coated NPs can be influenced by several factors, such as particle size, coating material, and the nature of the dopant ions. Figure 5a depicts the hysteresis loop (M–H) of the as-synthesized doped MFe_2_O_4_ NPs at room temperature, and the saturation magnetization (*M_s_*), coercivity (H_c_), and remanence (M_r_) values deduced from the loops are summarized in Table 2. From the results, it is evident that the polymer coat directly affects the coercivity, remanence, and saturation of the MFe_2_O_4_ NPs. Almost all coated ferrites behave as ferromagnetic, with small and almost negligible H_c_ (Figure 5a inset) and magnetic remanence values varying between M_r_ = 9.4 and 27.8 emu/g. This behavior is likely due to the strong ferromagnetic and anisotropic nature of the doped ions occupying octahedral sites. In such configurations, the spin–orbit coupling is insufficient to fully quench the orbital magnetic moment, leading to significant spin–orbit coupling effects and pronounced magnetic anisotropy [40,41,42]. Importantly, the *M_s_* magnetization obtained for PEG-CoFe_2_O_4_, PEG-NiFe_2_O_4_, PEG-ZnFe_2_O_4_, PVP-NiFe_2_O_4_, PVP-CoFe_2_O_4_NPs, and PVP-ZnFe_2_O_4_ ferrites were found to be equal to 65.87, 52.56, 37.75, 58.37, 47.29, and 20.94 emu/g, respectively. The highest saturation magnetizations were found for PEG- and PVP-coated Co- and Ni-doped samples, with the lowest obtained for Zn-doped ferrites. PVP-NiFe_2_O_4_ NPs showed slightly higher magnetization than PVP-CoFe_2_O_4_ NPs, probably due to crystallite size differences, as depicted by XRD Rietveld analysis (13.79 nm vs. 7.20 nm). Typically, and as observed by us and others, the *M_s_* values of MNPs decrease with a reduction in particle size, due to a reduction in magnetic anisotropy, while larger-sized MNPs show higher *M_s_* values [43,44]. This general trend in the magnetic properties of the doped ferrites is to some extent similar to that of earlier observations [42,45,46,47]. These differences in magnetic properties can be caused by several factors, including the nature of the polymer coat, the type of spinel ferrites, the ionic radii of the doped metals, and their distribution in the octahedral and tetrahedral sites, as well as the sizes of the nanocrystallites obtained [48]. Accordingly, the difference in site occupancies among the different ferrite crystal structures had a major influence on their magnetic properties [49,50,51]. Furthermore, in our XRD measurements, the Ni and Co ferrites exhibited more intense, sharper peaks than the Zn ferrites. Notably, however, both the PVP- and PEG-coated MFe_2_O_4_ ferrites synthesized in this study showed relatively higher saturation magnetization compared to similar polymer-coated ferrite NPs reported in the literature [37,52,53,54]. These findings suggest that the polymer-coated MFe_2_O_4_ ferrite NPs developed here possess enhanced magnetic properties, making them promising candidates for various applications.

To further confirm the unique magnetic behavior of the different doped ferrite samples, the effective anisotropy constant (K_eff_) was calculated, as it is one of the key parameters influencing magnetization. We used the law of approach to saturation (LAS) to determine K_eff_, applying the following equation to the experimental magnetization [55]:

$$M(H)=M_{s}\left(1-\frac{b}{H^{2}}\right)$$

where b is a parameter that is deduced from the fitting of experimental magnetization with the above equation. K_eff_ is then determined by the following:

$$K_{eff}={\mu_{0}M}_{s}\sqrt{\frac{15b}{4}}$$

As expected, the highest calculated K_eff_ values were found for the polymer-coated Co- and Ni-doped ferrite NPs (~2 × 10^5^ erg/cm^3^) (Table 2). Fitting the equation to the experimental magnetization shows that all of the obtained M-H curves fit well with the LAS model (Figure 5b).

### 3.3. Antibacterial Activity

**Figure 6:**
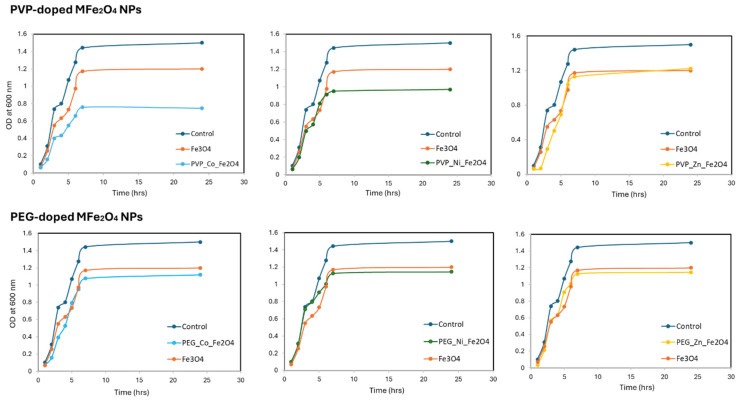
Growth curves of *E. coli* under the influence of different polymer-coated ferrite NPs compared to the normal growth curve of *E. coli*. The results clearly show that polymer-coated Ni- and Co-doped ferrites depict the highest antibacterial efficinecies, with superior effects for the PVP-Co-Fe_2_O_4_ ferrites.

Next, the antibacterial activities of the different polymer-coated MFe_2_O_4_ NPs against the Escherichia coli (*E. coli*) strain were investigated. The data presented in the growth curve analysis show the impact of the various MFe_2_O_4_ NPs on the growth and proliferation of the *E. coli* bacterial strain (Figure 6). A total of 25 mg of various metal-doped ferrites was incubated with 50 mL of the *E. coli* solutions (0.5 mg/mL) for different time points. The control growth curve demonstrates the typical growth pattern of *E. coli*, which follows a well-established trend. Initially, there is a lag phase where the bacteria adapt to the new environment and prepare for rapid growth. This is followed by an exponential growth phase, where the bacteria multiply at an increasing rate. Eventually, the growth reaches a stationary phase, where the population size stabilizes due to resource depletion and other limiting factors. This control growth curve serves as the baseline to assess the impact of our doped samples on bacterial growth and proliferation. By examining the growth curves of the *E. coli* cultures exposed to the various doped MNPs, it is evident that they all exhibit a distinct inhibitory effect on bacterial growth when compared to the control. This observation suggests that the MNPs, undoped or doped with the divalent metals, possess inherent antimicrobial properties that can effectively hinder the growth and proliferation of *E. coli*. The degree of inhibition, however, varies depending on the specific divalent metal and the polymer utilized. Undoped Fe_3_O_4_ NPs and ZnFe_2_O_4_ NPs showed very small effects. PVP-CoFe_2_O_4_ and PVP-NiFe_2_O_4_ NPs exhibited the most pronounced inhibitory antibacterial effects, as indicated by the consistently lower OD values compared to the control group. This observation suggests that the incorporation of Ni and Co ions into the MF_2_O_4_ NPs, along with PVP coating, synergically enhances their antimicrobial efficacy. PEG-coated doped MFe_2_O_4_ nanocomposites also demonstrated adequate antimicrobial activity, albeit slightly less than the PVP-doped counterparts.

**Figure 7:**
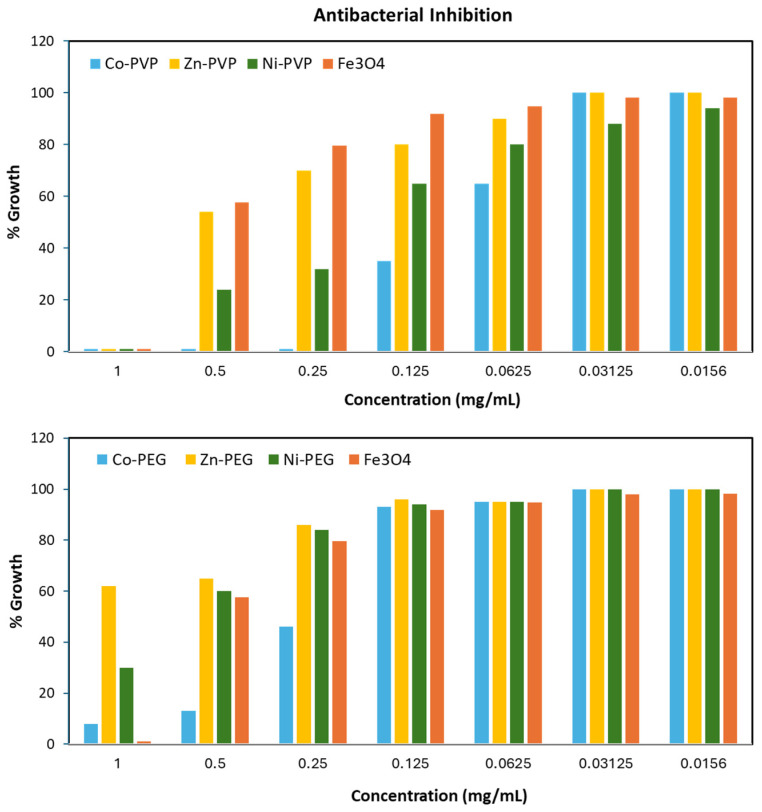
Bacterial growth percentages of *E. coli* in the presence of different concentrations of polymer-coated ferrite NPs. The results clearly show that polymer-coated Ni- and Co-doped ferrites depict the highest antibacterial efficinecies, with superior effects for the PVP-CoFe_2_O_4_ ferrites.

To study the effect of concentration on bacterial growth, the minimum inhibitory concentration (MIC) was studied using the microdilution method [56]. The results are outlined in Figure 7 and Table 3. As the results show, bacterial growth was significantly reduced in the presence of PVP-CoFe_2_O_4_ at concentrations ~62.5 µg/mL (>50% inhibition). No bacterial growth was observed at 250 µg/mL or higher concentrations, indicating an MIC_90_ of ~150–200 µg/mL. PVP-NiFe_2_O_4_ came next with slightly lower MICs. In the presence of PEG-CoFe_2_O_4_ and PEG-NiFe_2_O_4_, *E. coli* could grow at different concentrations, however, the growth was affected in concentrations of 250 µg/mL and above. No significant inhibition was found in the presence of PVP-ZnFe_2_O_4_ or PEG-ZnFe_2_O_4_, even at the highest concentrations (only at concentrations > 500 µg/mL were mild inhibition effects were found). In general, all of the doped MFe_2_O_4_ NPs showed better MICs, as compared to free undoped Fe_3_O_4_, similar to the earlier findings [23,27]. Collectively, these results demonstrated that both the metal doping and surface functionalization influenced the antimicrobial efficacy and performance of the ferrite NPs. In fact, the PVP-coated metal-doped ferrites exhibited improved antibacterial activity compared to the PEG-coated counterparts. The antibacterial performance was as follows: PVP-CoFe_2_O_4_ > PVP-NiFe_2_O_4_ > PVP-ZnFe_2_O_4_. The presence of the PVP polymer along with the divalent metal ions, particularly Co and Ni, resulted in the highest antibacterial inhibition and inactivation of the bacterial cells. It is to be noted that the bacterial cell walls of Gram-negative bacteria (i.e., *E. coli*) are much more complex than those of Gram-positive bacteria (possessing weaker cell walls and cell membranes) and are, therefore, less susceptible to antimicrobial agents [18]. Therefore, the doped ferrite NPs presented in this study serve as excellent alternative antimicrobial agents, since they exhibited considerable effects on Gram-negative bacterial strains.

The observed differences in antimicrobial efficacies of the divalent-metal-doped ferrites can be attributed to many factors and the varying abilities of the metal ions to interact with and disrupt the essential functions of the bacterial cells. The binding of metal ions to the sulfhydryl, amino, and carboxyl groups of bacterial proteins and enzymes can lead to the inactivation of critical cellular processes, contributing to the antimicrobial properties of these materials [29,57]. Specifically, the generation of ROS by MF_2_O_4_ NPs, particularly the Co- and Ni-doped variants, can induce oxidative stress and damage in the bacterial cells, further enhancing their antimicrobial efficacy [57,58]. In addition, the ultra-small sizes of the ferrite NPs, along with the coating, might be an additional important factor. This increases the permeability of the NPs to attach to the bacterial surface and enter *E. coli*. Smaller metal-doped NPs (20–200 nm) tend to exhibit enhanced antimicrobial activity due to their higher surface-to-volume ratio, facilitating greater interaction with bacterial cells [18,59]. Once inside, the divalent ions released by the NPs trigger the production of high amounts of ROS and cellular oxidative stress, which could lead to DNA damage, mitochondrial damage, nuclear breakdown, and disruptions in electron transport across the cell membrane. They can also impair the efflux pumps, in addition to the deactivation of enzymes responsible for resistance activity. Prominently, PVP functionalization may have further modulated antimicrobial activity by altering the surface properties, enhancing dispersibility, and, thus, better adhesion and interactions with the bacterial cells. While the ability of Ni^2+^ or Co^2+^ to interact with *E. coli* resulted in a bactericidal effect, the incorporation of Zn^2+^ ions affected, but to lesser extent, the antimicrobial inhibition against this specific *E. coli* strain. The exact reason for this is still not clear, but it might be attributed to the mechanism of action of Zn^2+^ ions, which may not be as effective at disrupting essential cellular functions or generating sufficient oxidative stress to inhibit bacterial growth as compared to Ni^2+^ or Co^2+^. All of these results suggest that the specific interactions and mechanisms of action of each metal ion with the specific *E. coli* strain may play a crucial role in determining the overall antimicrobial efficacy.

### 3.4. Cytotoxicity and Safety Profiles

**Figure 8:**
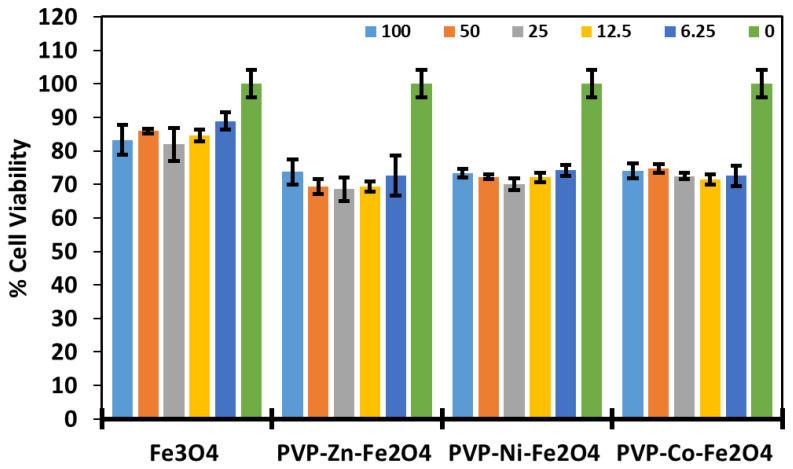
Percentage of viable HBF cells upon incubation with different concentrations of PVP-coated MFe_2_O_4_ NPs (0–100 μg/mL), as determined by MTT cell viability assay. The results clearly depict the low toxicity and biocompatibility of the doped ferrites, even at the highest concentrations of 100 µg/mL (~70% of the cells remained viable). The experiments were carried out in quadruplicates, and the error bars denote standard deviations. Data are presented as mean values ± standard deviations.

Before utilization of the as-prepared polymer-coated ferrite MFe_2_O_4_ NPs for biomedical applications, it is essential to assess their cytotoxicity, biocompatibility, and safety profiles. We focused on the PVP formulations with superior antibacterial effects (i.e., PVP-coated Ni, Co, and Zn-doped ferrites). Consequently, the cytotoxicity of various concentrations of the doped ferrites towards HBF human cell lines was evaluated using thiazolyl blue tetrazolium bromide (MTT) viability assay. As can be seen in Figure 8, both undoped and divalent-doped metal ferrites were not significantly toxic to the cells, even at considerably high concentrations of 100 µg/mL (~70% of the cells remained viable). This aligns with the standardized guidelines for the MTT assay, which defines toxicity as having less than 70% viability. Based on these results, we confirm safe doses of MFe_2_O_4_ ferrite NPs towards human cells, as most of the cells were still viable. These results corroborate well with previous studies testing the biocompatibility of doped ferrites [27,60], repeatedly confirming the safety profiles for magnetite and doped ferrites, where no significant potency was attained. Thus, combining the good biocompatibility profiles along with the antibacterial inhibition properties suggests that the fabricated PVP-coated doped ferrites hold great potential in areas such as water disinfection, biomedical devices, and antimicrobial applications.

The development of tailor-made divalentmetal-doped MF_2_O_4_ NPs, particularly PVP-coated Co- and Ni-doped NPs, as effective antimicrobial agents could contribute to the ongoing efforts in combating antibiotic-resistant bacterial infections and promoting improved infection control strategies. The potential of these nanomaterials to inhibit bacterial growth suggests their applicability in various fields, including healthcare, food processing, and water treatment. The observed results, herein, suggest that the strategic selection and incorporation of specific metal ions with appropriate coatings can be crucial factors in optimizing antimicrobial efficacies. By leveraging the unique properties of specific ferrites, researchers can explore the design and optimization of antimicrobial coatings, filters, or devices that can effectively mitigate the spread of bacterial infections and enhance the safety and reliability of various products and environments. Overall, the results demonstrate the potential of divalent-metal-doped ferrite NPs as a novel effective class of antimicrobial materials with tailorable properties and activities, offering promising avenues for further exploration and applications.

## 4. Conclusions

In conclusion, various PVP- and PEGylated metal-doped MFe_2_O_4_ NPs (M = Ni, Co, and Zn) were prepared using KHB methodology. It was shown that the doping of ferrites with various divalent metal ions, as well as coating them with different polymers, can influence the antimicrobial performance and efficacy of these NPs. For instance, the use of surface coatings like PVP or PEG can modulate the NP–cellular interactions and potentially improve the antimicrobial activities. The antimicrobial performance attained was as follows: PVP-CoFe_2_O_4_ > PVP-NiFe_2_O_4_ > PVP-ZnFe_2_O_4_. Thus, by leveraging the unique properties of each divalent-metal-doped ferrite NP, including their small sizes, polymer coating, and metal dopant, these nanomaterials can be developed as effective antimicrobial agents for various applications, such as water disinfection, biomedical devices, and antimicrobial coatings. Lastly, viability assays against human HBF cells confirmed the safety profiles of the utilized PVP–ferrites. The good antimicrobial properties and low toxicities of such PVPylated doped ferrite NPs strongly suggest their promising potential for various antimicrobial applications.

## Figures and Tables

**Table 1:** Rietveld analysis data.

Sample	Phase Composition (%)	Crystallite Size (nm)	Microstrain (%)	Lattice Parameters (Å)	Fit Parameters
PEG-CoFe_2_O_4_	CoFe_2_O_4_ 100	10.37	0.350	a = b = c = 8.369 α = β = γ = 90°	R_wp_ = 38.99% R_e_ = 20.20% R_p_ = 28.20% S = 1.0646 χ_2_ = 1.1334
PEG-NiFe_2_O_4_	NiFe_2_O_4_ 100	11.90	0.140	a = b = c = 8.379 α = β = γ = 90°	R_wp_ = 40.47% R_e_ = 36.71% R_p_ = 29.85% S = 1.1010 χ_2_ = 1.2122
PEG-ZnFe_2_O_4_	ZnFe_2_O_4_ 100	7.80	0.330	a = b = c = 8.404 α = β = γ = 90°	R_wp_ = 46.81% R_e_ = 45.38% R_p_ = 36.63% S = 1.0302 χ_2_ = 1.0614
PVP-CoFe_2_O_4_	CoFe_2_O_4_ 100	7.20	0.506	a = b = c = 8.431 α = β = γ =90°	R_wp_ = 37.39% R_e_ = 36.29% R_p_ = 27.02% S = 1.0288 χ_2_ = 1.0584
PVP-NiFe_2_O_4_	NiFe_2_O_4_ 100	13.79	0.228	a = b = c = 8.383 α = β = γ =90°	R_wp_ = 40.92% R_e_ = 37.56% R_p_ = 30.47% S = 1.0878 χ_2_ = 1.1833
PVP-ZnFe_2_O_4_	ZnFe_2_O_4_ 100	8.07	0.410	a = b = c = 8.407 α = β = γ = 90°	R_wp_ = 42.49% R_e_ = 39.74% R_p_ = 31.98% S = 1.0678 χ_2_ = 1.1401

R_wp_: Weighted profile R-factor; R_e_: Expected R-factor; R_p_: Profile R-factor; S: Goodness of fit (S = R_wp_/R_e_); χ_2_ = S^2^.

**Table 2:** Magnetic parameters deduced from M-H curves and law of approach to saturation.

Sample	Experimental				Law of Saturation	
	H_c_	M_r_	M_s_	M_r_/M_s_	M_s_	K_eff_
	(Oe)	(emu/g)	(emu/g)		(emu/g)	(erg/cm^3^)
PEG-CoFe_2_O_4_	0.810	14.91	65.87	0.226	68.06 ± 0.24996	2.02 × 10^5^
PEG-NiFe_2_O_4_	1.331	10.19	52.56	0.194	53.24 ± 0.02422	1.04 × 10^5^
PEG-ZnFe_2_O_4_	1.064	14.14	37.75	0.375	38.66 ± 0.03468	1.21 × 10^5^
PVP-CoFe_2_O_4_	0.687	16.37	47.29	0.346	50.12 ± 0.11923	2.07 × 10^5^
PVP-NiFe_2_O_4_	0.921	9.416	58.38	0.161	60.58 ± 0.37308	1.95 × 10^5^
PVP-ZnFe_2_O_4_	0.225	27.77	20.94	1.326	23.31 ± 0.19074	1.29 × 10^5^

**Table 3:** Bacterial growth percentage in the presence of different concentrations of polymer-coated ferrite NPs.

MNPs Concentration (µg/mL)	Bacterial Growth %						
	PVP-Co	PEG-Co	PVP-Zn	PEG-Zn	PVP-Ni	PEG-Ni	Fe_3_O_4_
1000	0.00	8.00	0.00	62.43	0.00	30.68	0.00
500	0.00	13.23	54.87	65.05	24.96	60.74	57.68
250	0.00	46.73	70.79	86.91	32.48	84.13	79.50
125	35.00	93.50	80.66	96.61	65.05	94.57	81.82
62.5	65.44	95.00	90.69	95.35	80.95	95.10	84.78
31.3	100.00	100.00	100.00	100.00	86.77	100.00	98.07
15.6	100.00	100.00	100.00	100.00	100.00	100.00	98.22

## Data Availability

The original contributions presented in this study are included in the article. Further inquiries can be directed to the corresponding author.
